# The complete mitochondrial genome of *Ramulus bifarius* (Phasmida: Phasmatidae; Clitumninae)

**DOI:** 10.1128/mra.00359-26

**Published:** 2026-07-27

**Authors:** Rui Han, Xun Bian, Bin Zhang

**Affiliations:** 1 College of Life Sciences and Technology, Inner Mongolia Normal University 71203 , Hohhot, China; 2 Key Laboratory of Ecology of Rare and Endangered Species and Environmental Protection (Guangxi Normal University), Ministry of Education 12388, Guilin, China; 3 Key Laboratory of Biodiversity Conservation and Sustainable Utilization for College and University of Inner Mongolia Autonomous Region, Hohhot, China; Rochester Institute of Technology, Rochester, New York, USA

**Keywords:** mitogenome, China, *Ramulus*

## Abstract

We successfully acquired the complete mitochondrial genome information of *Ramulus bifarius* (S. C. Chen and Y. H. He, p. 476, *Phasmatodea of China*, 2008), which can be used for subsequent related molecular studies. The mitogenome of *R. bifarius* exhibits a circular duplex structure, with a total length of 16,915 bp and a high adenine and thymine bias of 76.7%. It contains 13 protein-coding genes, 22 tRNA genes, 2 rRNA genes, and a single control region.

## ANNOUNCEMENT

*Ramulus bifarius* is a species of walking stick belonging to the genus *Ramulus*, family Phasmatidae (1, 2). The genus *Ramulus* possesses unique mimicry abilities, which enable them to blend well into the surrounding environment to avoid natural enemies (3). The species *Ramulus bifarius* is distributed in Guangxi, China. Current knowledge of this species is restricted to morphological descriptions, with no in-depth molecular research previously reported. Accordingly, we sequenced its mitochondrial genome and performed comprehensive genomic analyses in the present study.

In this paper, the samples of *Ramulus bifarius* were collected from residential areas surrounding Mao'er Mountain, Guilin, Guangxi, in 2022 (25.882719°N 110.490609°E). The specimens were stored at 4°C in a refrigerator and deposited at Guangxi Normal University. After sample preservation, genomic DNA was extracted from the muscle of the hind femur using the TIANamp Genomic DNA Kit. High-throughput sequencing was performed by Beijing Berry Genomics Co., Ltd. A 150 bp paired-end library was constructed using the MGIEasy Library Preparation Kit (MGI) and subjected to high-throughput sequencing on the Illumina NovaSeq 6000 platform (Illumina Inc.). Following sequencing, the raw reads underwent quality control and preprocessing with fastp v.0.20.0 (4) to remove adapter sequences and primer residues, discard reads with a Phred quality score < Q5, and filter out reads containing more than three ambiguous (N) bases. The resulting clean reads were assembled into the complete mitochondrial genome using NOVOPlasty v4.2.1 (5), with *Entoria formosana* (GenBank accession no. PP536074.1) designated as the reference sequence and customized parameter settings: assembly type = mito, genome range = 14,000–18,000, k-mer = 33, max memory = 16, extended log = 0 (6–8); all remaining parameters were retained at their default values. The assembled mitochondrial genome had an average effective sequencing depth of 126×. Then, clean sequencing reads were deposited in the Sequence Read Archive (SRA) database for public access and re-evaluation. Finally, mitochondrial genome annotation was implemented on the Geneious Prime v 2026.0.2 web server.

**Fig 1:**
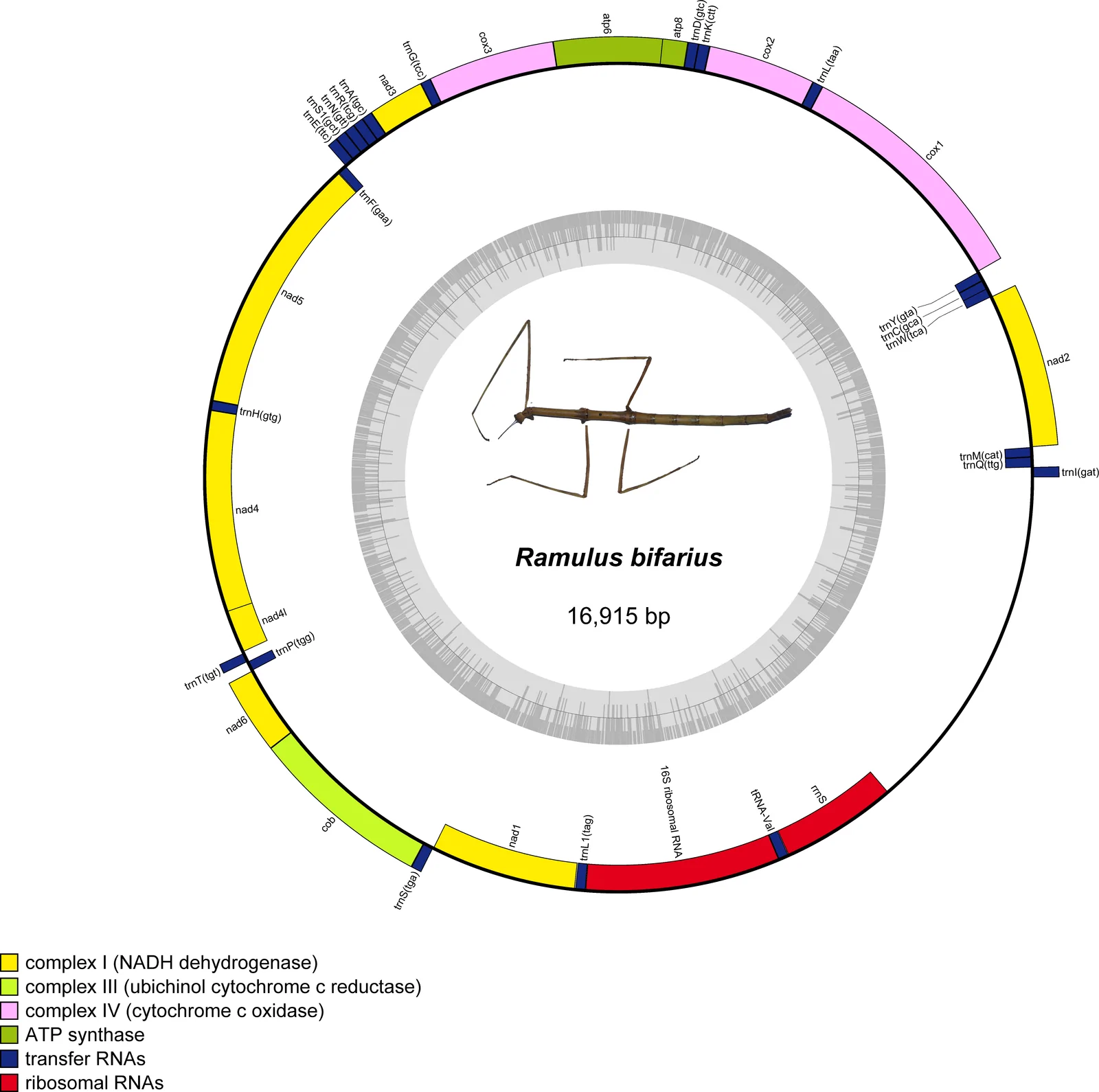
This circular map was generated using OrganellarGenomeDRAW v.1.3.1 (10), covering the image of *Ramulus bifarius*, all tRNA genes, protein-coding genes, rRNA genes, and simultaneously displaying the GC content characteristics.

As can be seen from Fig. 1, the mitochondria of *Ramulus bifarius* (GenBank accession no. PZ143327.1) exhibit a double-stranded circular structure with a total length of 16,915 bp. Its gene composition includes 13 protein-coding genes, 22 tRNA genes (tRNAs), 2 rRNA genes (rRNAs), and a control region. All 13 protein-coding genes utilize ATN as the initiation codon, with TAA and incomplete T serving as the termination codons. The transcription of TA or T may involve poly(A) tail addition to compensate for the deficiency and ensure translation termination (9). A total of 22 tRNA genes were annotated, with their lengths ranging from 62 bp (*trnT*) to 70 bp (*trnK*). The rRNA genes consist of *rrnS* (769 bp) and *rrnL* (1,287 bp), representing the two core ribosomal RNA components of the mitogenome (Table 1).

**TABLE 1:** Annotation and gene organization of the *R. bifarius*

Name	Location	Length	Start/stop codon	Direction
*trnI(gat)*	1–67	67	–^*a*^	Forward
*trnQ(ttg)*	65–133	69	–	Reverse
*trnM(cat)*	133–197	65	–	Reverse
*nad2*	198–1214	1,017	ATT/TAA	Forward
*trnW(tca)*	1213–1278	66	–	Reverse
*trnC(gca)*	1271–1333	63	–	Reverse
*trnY(gta)*	1334–1397	64	–	Reverse
*cox1*	1399–2932	1,534	ATG/T	Forward
*trnL(taa)*	2933–2996	64	–	Forward
*cox2*	2997–3666	670	ATA/T	Forward
*trnK(ctt)*	3667–3736	70	–	Forward
*trnD(gtc)*	3736–3801	66	–	Forward
*atp8*	3802–3960	159	ATA/TAA	Forward
*atp6*	3954–4631	678	ATG/TAA	Forward
*cox3*	4631–5419	789	ATG/TAA	Forward
*trnG(tcc)*	5419–5481	63	–	Forward
*nad3*	5482–5833	352	ATT/T	Forward
*trnA(tgc)*	5834–5899	66	–	Forward
*trnR(tcg)*	5900–5965	66	–	Forward
*trnN(gtt)*	5966–6032	67	–	Forward
*trnS1(gct)*	6033–6100	68	–	Forward
*trnE(ttc)*	6101–6166	66	–	Forward
*trnF(gaa)*	6167–6230	64	–	Reverse
*nad5*	6232–7954	1,723	ATT/T	Reverse
*trnH(gtg)*	7955–8018	64		Reverse
*nad4*	8018–9349	1,332	ATG/TAA	Reverse
*nad4l*	9343–9627	285	ATA/TAA	Reverse
*trnT(tgt)*	9636–9697	62	–	Forward
*trnP(tgg)*	9698–9762	65	–	Reverse
*nad6*	9764–10237	474	ATT/TAA	Forward
*cob*	10237–11368	1,132	ATG/T	Forward
*trnS(tga)*	11369–11436	68	–	Forward
*nad1*	11437–12400	964	ATA/T	Reverse
*trnL1(tag)*	12404–12470	67	–	Reverse
*rrnL rRNA*	12471–13757	1,287	–	Reverse
*trnV(tac)*	13758–13825	68	–	Reverse
*rrnS rRNA*	13827–14595	769	–	Reverse

^*a*^ –, not applicable.

## Data Availability

The complete mitochondrial genome sequence of *Ramulus bifarius* is available in GenBank under the accession number PZ143327.1. The corresponding BioProject, BioSample, and SRA numbers are PRJNA1429031, SAMN55977729, and SRR36997471, respectively.
